# Treatment of neuroendocrine tumours with infusional 5-fluorouracil, folinic acid and streptozocin

**DOI:** 10.1038/sj.bjc.6601167

**Published:** 2003-07-29

**Authors:** M A Gonzalez, S Biswas, L Clifton, P G Corrie

**Affiliations:** 1 Oncology Centre (Box 193), Addenbrooke's Hospital, Hills Road, Cambridge CB2 2QQ, UK; 2 MRC Cancer Cell Unit, Hutchison/MRC Research Centre, Cambridge CB2 2XZ, UK

**Keywords:** neuroendocrine tumour, carcinoid, streptozocin, 5-fluorouracil, LV5FU2

## Abstract

A standardised, effective systemic therapy for metastatic neuroendocrine tumours (NETs) has not been established to date. We reviewed the management of 15 patients with inoperable, metastatic NET treated systematically with a combination chemotherapy regimen of infusional 5-fluorouracil, folinic acid and streptozocin. Overall objective response rate was 53% and tolerability was excellent.

Neuroendocrine tumours (NETs) are very rare cancers with characteristically indolent behaviour. The mainstay of treatment is surgical resection. However, many tumours metastasise and cannot be removed, while their behaviour changes to become more aggressive over time such that the median life expectancy is approximately 2 years. A range of therapeutic modalities are available for the treatment of metastatic disease, but none has been shown convincingly to improve survival in randomised trials. Treatments offered to patients depend on whether the disease is hormonally functional and include manipulation with somatostatin analogues, radioisotope therapy and hepatic arterial embolisation (Caplin *et al*, 1998). Despite their low growth rate, NETs are considered to be chemosensitive and responses have been reported with a range of cytotoxic agents, in particular, streptozocin, 5-fluorouracil (5FU), doxorubicin, cisplatin and etoposide (Moertel *et al*, 1980, 1992; Rivera and Ajani, 1998; Cheng and Saltz, 1999; Fjallskog *et al*, 2001).

It has proved difficult to perform high-quality, adequately powered clinical trials in this rare cancer and few exist in the literature. In 1980, Eastern Cooperative Oncology Group (ECOG) published the results of a trial that randomised 84 NET patients to receive streptozocin chemotherapy or streptozocin combined with bolus injection of 5FU (Moertel *et al*, 1980). Combination therapy achieved a response rate of 63% compared with 36% for single-agent streptozocin. Streptozocin plus 5FU was subsequently widely adopted as a standard regimen for treating metastatic NETs. Since then, only two other chemotherapy regimens have been shown to rival this level of antitumour activity. The results of another ECOG trial (Moertel *et al*, 1992) suggested that doxorubicin was superior to 5FU when combined with streptozocin, in terms of response rate (69 *vs* 45%) and survival (median 2.2 *vs* 1.4 years). However, a retrospective analysis of 6 years of treating patients with NETs with streptozocin plus doxorubicin performed at the Memorial Sloane Kettering Cancer Center failed to confirm the ECOG trial results, with only one of 16 patients (6%) treated during that period achieving an objective radiological response (Cheng and Saltz, 1999). One of the reasons for the discrepancy in response rates between these two patient groups is that the ECOG trials included a biochemical response category in addition to conventional objective tumour reduction in size. Others have done likewise, and the response rate of 55% achieved by treating 36 patients in Sweden with cisplatin plus etoposide included nine biochemical responses (Fjallskog *et al*, 2001). Using conventional WHO criteria for measurable disease, the response rate was, in fact, 36%.

All three regimens described here are associated with considerable grade III/IV toxicity, in particular, myelosuppression. From studies primarily in colorectal cancer, it is clear that 5FU administered as an infusion with or without folinic acid (FA) is better tolerated and achieves higher response rates compared to bolus administration. We hypothesised that the standard streptozocin plus bolus 5FU chemotherapy regimen could be improved in terms of both cytotoxicity and patient tolerance by using an infusional 5FU regimen well established in colorectal cancer management (de Gramont *et al*, 1997). Therefore, we adapted the standard de Gramont regimen (also known as ‘LV5FU2’ (De Gramont *et al*, 1988) to include streptozocin when offering chemotherapy to patients with metastatic NETs. Here, we review the results of treating 15 patients with this regimen.

## PATIENTS AND METHODS

We analysed retrospectively the treatment of patients with unresectable NETs, treated in our centre with infusional 5FU, FA and streptozocin (‘FUFAS’) chemotherapy between January 1997 and December 2002. Histological confirmation of the diagnosis was obtained in all cases. The chemotherapy regimen comprised a 2-h infusion of FA (200 mg m^−2^), then a bolus injection of 5FU (400 mg m^−2^), followed by a 22 h infusion of 5FU (600 mg m^−2^), commenced on day 1 and repeated on day 2. Streptozocin (600 mg m^−2^, maximum dose 1 g) was administered on day 2 as a 30-min infusion. The regimen was repeated every 2 weeks. The criteria for commencing systemic chemotherapy were: (1) evidence of rapidly progressive disease on serial CT scan imaging, (2) extensive hepatic involvement or (3) symptomatic disease in hormonally functional tumours despite optimal treatment with a somatostatin analogue. Patients had CT evidence of measurable disease performed within 4 weeks of commencing treatment and response was determined independently by consultant members of the local radiology department, by repeat CT scanning after every six cycles, using conventional WHO response criteria. The worst toxicity was recorded for every cycle using NCI Common toxicity criteria.

## RESULTS

A total of 15 patients (eight male, seven female median age 60 years; range 27–68 years) with metastatic, nonpulmonary NETs were treated. The primary site of the tumour was the pancreas in six cases, the ovary in one case and unknown in eight patients. Liver involvement at presentation was evident in 12 (80%) patients. Four patients had secretory symptoms and had previously been challenged with a somatostatin analogue. The median number of cycles of chemotherapy received was six (range 1–14).

One patient achieved a complete response to treatment, seven patients demonstrated a partial response, two patients had stable disease and five had progression as best response. Overall objective response rate was therefore 53%. The median time to progression was 4 months for all patients and 12 months for responders. Of the eight responding patients, five had tumours originating in the pancreas. The overall survival at median follow-up of 27 months has not yet been reached and eight patients remain alive. No grade III/IV toxicities occurred. Gastrointestinal toxicities were the most frequently encountered (WHO Grade II diarrhoea in four patients). No patient required dose modification or treatment delays.

## DISCUSSION

The ECOG trials combining 5FU bolus injection with streptozocin indicated a response rate of 63 and 45%, with median survival of 17 months in both cases (Moertel *et al*, 1980, 1992). Since optimal administration of 5FU is now considered to be by infusion, we combined streptozocin with the De Gramont infusional 5FU regimen, which was well tolerated overall. In our, albeit small series, efficacy is at least as good as the ECOG data and this regimen warrants testing further in prospective trials. A UK centre with a specialist interest in NETs recently reviewed their experience of administering combination chemotherapy over a 22-year period, when 31 patients were treated (Kaltsas *et al*, 2002). However, 23 of these patients received therapeutic modalities in addition to chemotherapy. Thus, our study is important for being one of the largest groups of NET patients managed systematically with a single treatment regimen.

Owing to the low prevalence of NETs, controlled trials are difficult to perform. Even so, given the wide range of therapeutic modalities available, many of which have significant costs to both patients and the health service, evidence is required to better define their roles in the overall management of this disease. Larger scale trials are therefore warranted, which would also allow coordination of tissue collection for research into this tumour, which is currently poorly understood. For example, genomic and proteomic studies might serve as prognostic and predictive tools and assist in therapeutic decisions, as well as providing potential molecular targets for novel drug development.
